# Effects of Knee Sleeve Density on Theoretical Neuromuscular Capacities Derived from the Force–Velocity–Power Profile in the Back Squat

**DOI:** 10.3390/jfmk11010047

**Published:** 2026-01-22

**Authors:** Jorge Leschot-Gatica, Luis Romero-Vera, Alberto Ñancupil-Andrade, Claudio Hernández-Mosqueira, Iván Molina-Márquez, Rodrigo Yáñez-Sepúlveda, Felipe Montalva-Valenzuela, Eduardo Guzmán-Muñoz

**Affiliations:** 1 Facultad de Salud y Ciencias Sociales, Universidad de Las Américas, Licenciatura en Ciencias de la Actividad Física, Concepción 4030000, Chile; jleschotg@gmail.com; 2 Department of Sports Sciences and Physical Conditioning, Universidad Católica de la Santísima Concepción, Concepción 4030000, Chile; luis.romero@ucsc.cl; 3 Department of Health, University of Los Lagos, Puerto Montt 5400000, Chile; 4 Departamento de Ciencias de la Educación, Universidad del Bio-Bio Bio, Chillan 3780000, Chile; chernandez@ubiobio.cl; 5 Escuela de Educación Física, Facultad de Educación, Universidad Adventista de Chile, Chillan 3780000, Chile; ivanmolina@unach.cl; 6 Programa Doctorado en Ciencias de la Actividad Física, Universidad Católica del Maule, Talca 3460000, Chile; 7 Faculty Education and Social Sciences, Universidad Andrés Bello, Viña del Mar 2520000, Chile; rodrigo.yanez.s@unab.cl; 8 School of Medicine, Universidad Espíritu Santo, Samborondón 092301, Ecuador; 9 Escuela de Entrenador en Actividad Física y Deporte, Facultad de Ciencias Humanas, Universidad Bernardo O’Higgins, Santiago 8370040, Chile; felipemontalva95@gmail.com; 10 Escuela de Kinesiología, Facultad de Salud, Universidad Santo Tomás, Talca 3460000, Chile; eguzmanm@santotomas.cl; 11 Pedagogía en Educaciόn Física, Facultad de Educaciόn, Universidad Autόnoma de Chile, Talca 3460000, Chile

**Keywords:** CMJ, ergogenic tool, muscular performance, powerlifting, resistance training, vertical jump, resistance training, muscle strength, athletic performance, force–velocity relationship

## Abstract

**Background**: Neoprene knee sleeves are commonly used to enhance joint stability and mechanical performance during resistance training. However, the specific influence of sleeve density on the force–velocity–power (F–V–P) profile during multi-joint lower-body exercises such as the back squat remains unclear. This study aimed to compare the theoretical F–V–P parameters derived from back squat performance while wearing low-density (LD) versus high-density (HD) knee sleeves. **Methods**: Fifteen resistance-trained males completed an incremental back squat test under both LD and HD conditions. A linear position transducer recorded barbell displacement and velocity. Individual force–velocity relationships were modelled to determine maximal theoretical force (F_0_), velocity (V_0_), power (Pmax), and the F–V slope. Paired-sample t-tests, linear mixed models, and Cohen’s d effect sizes were calculated. Clinical relevance was assessed using a threshold defined as 0.2 × the standard deviation of the HD condition. Bayesian analyses were conducted to estimate the probability and magnitude of the observed effects. **Results**: No statistically significant differences were observed between sleeve conditions for F_0_, V_0_, Pmax, or F–V slope (*p* > 0.05, d ≤ 0.37). Nonetheless, HD sleeves yielded slightly higher mean values for F_0_, V_0_, and Pmax, exceeding the predefined threshold for practical relevance. Bayesian models showed moderate probabilities (~0.80) that HD sleeves outperformed LD, though with limited chances of crossing the clinical significance threshold. **Conclusions**: Although HD sleeves do not produce systematic changes in F–V–P parameters, their increased material stiffness may provide small yet practically meaningful mechanical advantages in high-force resistance training contexts.

## 1. Introduction

Strength training is widely recognised as a key element in optimising neuromuscular performance [1,2,3], particularly in compound movements such as the squat, where strength, speed and power interact to determine overall performance [4,5,6]. Evidence suggests that powerlifting-based training programmes, when implemented in different sports disciplines, can significantly improve physical performance [7], underscoring the relevance of this training model not only within powerlifting itself, but also in broader contexts of physical conditioning and athletic development [8]. At the same time, the use of orthopaedic and support devices has received increasing attention in strength training due to their potential ergogenic benefits [9,10]. These benefits include improved performance and injury prevention in multiple strength sports, such as CrossFit, powerlifting, strongman, bodybuilding and Olympic weightlifting [11,12]. Among these, powerlifting is particularly notable for its emphasis on maximum strength, which is assessed through three main lifts: squat, bench press, and deadlift, with athletes allowed up to three attempts per exercise [13]. Proper execution of a barbell squat requires significant knee flexion and extension, which subjects the joint to considerable mechanical stress [14,15]. To mitigate this stress and potentially improve performance, athletes often use external supports such as bandages or neoprene knee pads [16].

These devices can influence performance through several proposed mechanisms. Specifically, (a) improved proprioceptive feedback and reduced muscle oscillation, as described in the literature on compression and support garments [17,18,19,20], and (b) the ability of passive tissues to store and release elastic energy during the eccentric and concentric phases of the squat [21]. These mechanisms have been supported by biomechanical studies on compression garments and neoprene aids, which suggest that such equipment can improve the efficiency of vertical force transmission during the lifting phase [22,23]. However, the available evidence on performance enhancement associated with the use of neoprene knee braces in resistance training remains ambiguous [9,24]. While there are limited data comparing performance outcomes with different knee brace densities, a recent study evaluated performance in barbell squats and counter-movement jumps (CMJ) using low-density (LD) and high-density (HD) knee braces [25]. The authors reported an increase in one-rep maximum (1RM) during the barbell squat with HD, while no clear effects on CMJ performance were observed [25]. These preliminary findings underscore the need for further research exploring the biomechanical and neuromuscular influences of knee pad density, particularly in strength-oriented tasks [22,23,24,25].

Despite the widespread use of knee braces in strength training and competitive settings, little is known about how knee brace density modulates underlying parameters of neuromuscular performance [19,26]. Traditional outcome measures, such as jump height or 1RM, offer limited insight into the mechanical determinants of performance [27,28]. In contrast, the force–velocity–power profile approach offers a more comprehensive characterisation of muscle function by quantifying force production, movement velocity, and power output across a spectrum of loads [29,30]. To date, no scientific literature has been reported that has explored the effects of knee brace density using this integrative framework, which could reveal subtle adaptations in performance that go unnoticed with conventional testing methods [31].

Therefore, the aim of this study was to compare maximum neuromuscular capacities (i.e., theoretical maximum force (F_0_), theoretical maximum velocity (V_0_), maximum power output (P_max_), and the slope of the force–velocity relationship) between low- and high-density knee pads during squat performance. It was hypothesised that the high-density knee brace would generate greater strength and power results due to its greater mechanical rigidity.

## 2. Materials and Methods

### 2.1. Study Sample and Design

A descriptive and comparative cross-sectional design was used for this investigation. The assessments took place at a sports facility in Concepción, Chile. Ethical approval was granted by a local Institutional Ethics Committee. All procedures adhered to the ethical standards set forth in the Declaration of Helsinki [32]. Prior to data collection, participants were thoroughly informed—both in writing and verbally—about the study’s objectives, procedures, potential risks, and benefits. Written informed consent was obtained from all participants, who were also advised of their right to withdraw from the study at any point without consequences.

A total of fifteen male participants (aged 25.13 ± 2.39 years), all with prior resistance training experience, were recruited using a convenience sampling method based on availability. To be eligible, individuals had to meet the following criteria: (a) a minimum of one year of continuous resistance training with a frequency of at least three sessions per week, and (b) the ability to perform a one-repetition maximum (1RM) back squat equivalent to or greater than 1.5 times their body mass. Participants were excluded if they (a) had used anabolic steroids within the past 12 months or (b) had sustained any musculoskeletal injury in the 12 months preceding the study.

Descriptive statistics for the 15 male participants are presented in Table 1. Overall, the sample consisted of young, recreationally trained men with relatively homogeneous anthropometric characteristics in terms of age, body mass, height, and body mass index (BMI). All participants met the inclusion criteria for training experience and strength level, forming a cohort representative of resistance-trained individuals typically involved in back-squat-based training.

Prior to conducting the physical performance tests, participants completed a structured questionnaire designed to capture sociodemographic and training-related data, including age, duration of strength training experience, training frequency, nutritional practices, and the use of performance-enhancing substances. Information regarding health status, such as existing comorbidities and history of musculoskeletal injuries, was also collected. Anthropometric measurements included body mass, assessed using a digital scale (Seca 803, Seca GmbH, Hamburg, Germany), and standing height, measured with a stadiometer (Seca 217, Seca GmbH, Hamburg, Germany).

Data collection was conducted over a six-month period between January and June 2024.

**Table 1:** Descriptive statistics for participant characteristics. Values are presented as mean and SD, as well as minimum and maximum observed values.

	Mean	SD	Max	Min
**Age (years)**	25.13	2.39	30	22
**Weight (kg)**	78.60	6.28	92.00	70
**Height (m)**	1.73	0.07	1.93	1.65
**BMI (k** **g/m^2^)**	26.70	2.23	31.83	23.41

Abbreviations: SD: Standard deviation, kg: Kilograms, m: metres, BMI: body mass index.

### 2.2. Procedure

The study protocol consisted of one familiarisation session followed by two experimental trials, with each session separated by 48 to 72 h to allow sufficient recovery. During the familiarisation phase, participants completed an incremental free-weight back squat assessment without the use of knee sleeves. The protocol began at 50% of the participant’s self-reported 1RM and increased in load until the barbell velocity approached approximately 0.5 m/s, a value associated with about 85% of maximum intensity [33], as monitored by a linear position transducer.

At the beginning of the first experimental trial, participants were randomly allocated to begin with either the low-density (LD) or high-density (HD) knee sleeve condition. Allocation was carried out using a simple random draw method, where each participant selected a slip of paper from an opaque container. The condition not selected during the first trial was automatically assigned for the second, ensuring a balanced crossover design.

Due to the observable physical differences between the sleeves—such as material stiffness and thickness—blinding was not possible. As a result, both the participants and researchers were aware of the sleeve condition in each session, which is acknowledged as a methodological limitation.

To maintain consistency and ecological validity, all participants wore the same footwear and used the same training equipment throughout the study. They were instructed to follow their customary lower-body warm-up routines prior to testing. The testing procedures remained identical across all sessions to minimise the risk of procedural bias. Additionally, during both the familiarisation and experimental sessions, real-time velocity feedback was provided, and participants were consistently encouraged to execute each repetition with maximal intent [34].

### 2.3. Back Squat Assessment

Maximal back squat performance was assessed through an incremental loading protocol while continuously tracking mean velocity (MV) using a linear position transducer (Chronojump, Barcelona, Spain) attached to the barbell. The system provided real-time velocity feedback and exported repetition-level mean concentric velocity (m/s) at each load. In addition, repetition-level estimates of mean force (N) and mean power (W) were obtained for subsequent force–velocity–power profiling. Testing commenced at 50% of the participant’s self-reported one-repetition maximum (1RM), with subsequent load increments tailored to the bar velocity: increases of 40 kg for MV ≥ 1.00 m/s, 30 kg for ≥0.70 m/s, 20 kg for ≥0.60 m/s, 10 kg for ≥0.50 m/s, 5 kg for ≥0.40 m/s, and 2.5 kg for ≥0.35 m/s.

The protocol continued until concentric failure was reached, operationalized as the inability to complete two consecutive repetitions at a given load. The final successful repetition prior to failure was designated as the participant’s 1RM. Rest periods between sets ranged from 1 to 5 min, adjusted according to the relative intensity of the load. Participants were instructed to perform up to six repetitions at lighter loads and to limit efforts to a single repetition at higher intensities.

### 2.4. Data Processing and Construction of the Force–Velocity–Power Profile

Data were collected through an incremental back squat protocol performed under two experimental conditions: using low-density (LD) and high-density (HD) knee sleeves. Each participant completed multiple repetitions with progressively heavier loads. For each repetition, mean concentric velocity (v, in m/s), mean force (F, in N), and mean power (*p*, in W) were recorded. The data were organised into a long-format data frame, allowing detailed analysis by load and individual subject.

### 2.5. Calculation of Theoretical Neuromuscular Capacities

The use of a linear model to estimate F_0_, V_0_, and Pmax is widely supported in the scientific literature as a valid and sensitive approach to assess neuromuscular performance in submaximal efforts [35,36]. Additionally, LOESS (locally estimated scatterplot smoothing) has been recommended in applied research as an effective exploratory technique for depicting overall trends when variable relationships show local dispersion or slight nonlinearity [37,38]. Combining both approaches enabled a robust and visually interpretable description of the group-level force–velocity–power profile.

For each participant and under each experimental condition (LD and HD), a simple linear model was fitted with force as the dependent variable and velocity as the independent variable (F ~ v). From this model, the following theoretical neuromuscular capacities were derived: F_0_ (theoretical maximal force), estimated as the model intercept corresponding to the force value when velocity equals zero; V_0_ (theoretical maximal velocity), calculated as the ratio of the intercept to the slope, representing the theoretical velocity when force is zero; and Pmax (theoretical maximal power), obtained as (F_0_ × V_0_)/4, which corresponds to the optimal point of power production under the linear model.

This procedure was applied to all participants in both conditions, resulting in individual estimates of their neuromuscular capacities for subsequent comparison.

### 2.6. Statistical Analysis

Statistical analyses were performed using RStudio (R version 2024.12.1+563, R Foundation for Statistical Computing, Vienna, Austria). Descriptive statistics were calculated for all variables. Depending on the distribution, results are presented as mean ± standard deviation (SD) for normally distributed variables or median and interquartile range (IQR) for non-normally distributed variables.

Normality of distributions was assessed with the Shapiro–Wilk test. For normally distributed variables, paired Student’s *t*-tests were applied to compare LD and HD conditions. To account for the repeated-measures structure, linear mixed-effects models were also fitted, with condition (LD vs. HD) as a fixed effect and subject as a random effect.

Effect sizes were calculated as Cohen’s *d* for paired designs and interpreted as trivial (<0.20), small (0.20–0.49), moderate (0.50–0.79), or large (≥0.80). To complement statistical significance, we also evaluated clinical significance by defining a minimum meaningful threshold of 0.2 × SD of the HD condition for each outcome.

To visually represent the force–velocity and power–velocity profiles for both conditions, a local regression smoothing was applied using the LOESS method on data aggregated by condition.

The analysis was performed on the complete set of observations for each condition, without separating by individual subjects, in order to represent the average group behaviour. A span value of 0.75 was empirically selected after testing various configurations, as it provided an optimal balance between smoothness and fidelity to the raw data trend. The smoothing adjustment was implemented using the semismooth (method = “loess”, span = 0.75) function from the ggplot2 package (v4.0.1) in R (v4.5.2), and the resulting curve was visually validated by overlaying it with the raw data to ensure consistency and the absence of smoothing artefacts. These curves allowed for visual inspection and comparison of LD and HD conditions regarding the force–velocity and power–velocity relationships, supplementing the numerical analysis of individual capacities.

In addition to the frequentist analyses, Bayesian procedures were conducted to quantify the strength and practical meaning of the observed effects. First, paired-sample Bayesian t-tests (JZS prior, r = 0.707) were computed for F_0_ and Pmax to estimate Bayes factors (BF_10_) comparing the alternative hypothesis (HD ≠ LD) against the null. Second, Bayesian linear mixed-effects models were fitted using the *brms* package (v2.23.0), with condition as a fixed effect and subject as a random effect, mirroring the structure of the frequentist models. Posterior distributions were summarised through estimates, 95% credible intervals, and effective sample sizes. To assess practical relevance, the posterior probability of the HD condition exceeding the clinical threshold of 0.2 × SD (HD) was calculated for each variable, together with the probability of a positive effect (HD > LD) and the probability that the effect fell within the region of practical equivalence (±0.2 × SD). These Bayesian analyses provided complementary evidence by quantifying uncertainty and estimating the likelihood of meaningful effects beyond traditional significance testing.

## 3. Results

**Figure 1:**
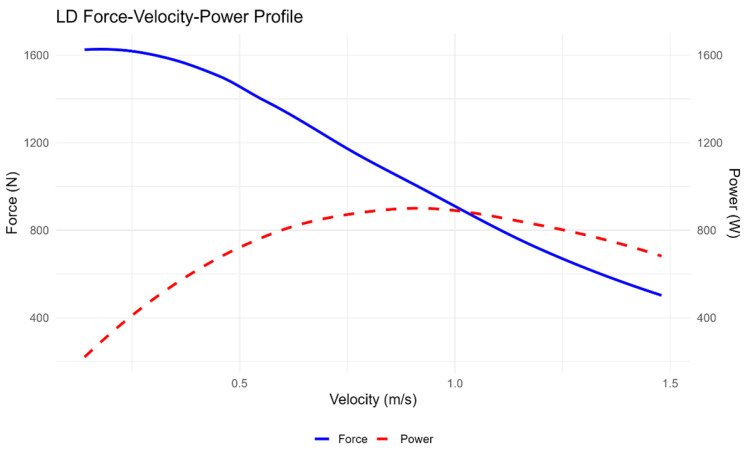
LD Force–velocity–power profile. Average force (N, left Y-axis) and power (W, right Y-axis) values are plotted against movement velocity (m/s) under the low-density (LD) knee sleeve condition. Each curve represents a second-degree polynomial fit based on the group mean values. The force–velocity relationship displays the expected inverse linear trend, while the power–velocity curve demonstrates a parabolic profile, with peak power observed at intermediate velocities. This profile illustrates the typical mechanical behaviour of lower-limb power output and can be used to inform individualised load-velocity training strategies.

**Figure 2:**
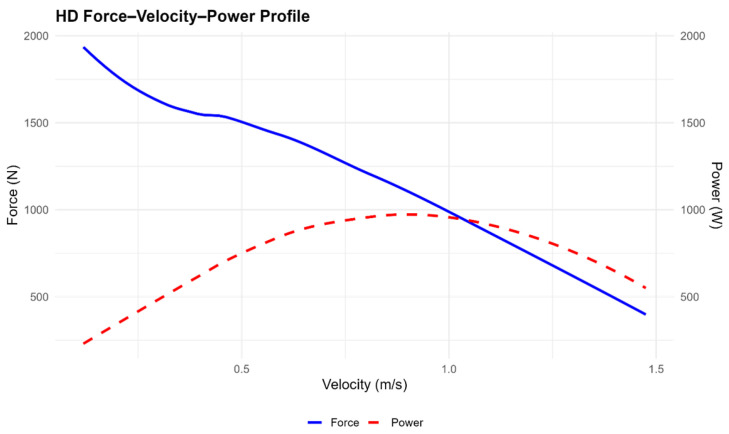
HD Force–Velocity–Power Profile. Mean group profiles of force (N), power (W), and velocity (m/s) are shown for the high-density (HD) knee sleeve condition. The left Y-axis represents force output, while the right Y-axis corresponds to power. Velocity is represented on the X-axis. Each curve reflects a second-degree polynomial fit to the averaged data across all participants. The force–velocity profile demonstrates the expected inverse relationship, while the power–velocity curve displays a parabolic pattern, with peak power occurring at intermediate velocities. These trends provide insight into mechanical output under HD compression and can assist in evaluating load–velocity–power characteristics during squat-based movements.

**Figure 3:**
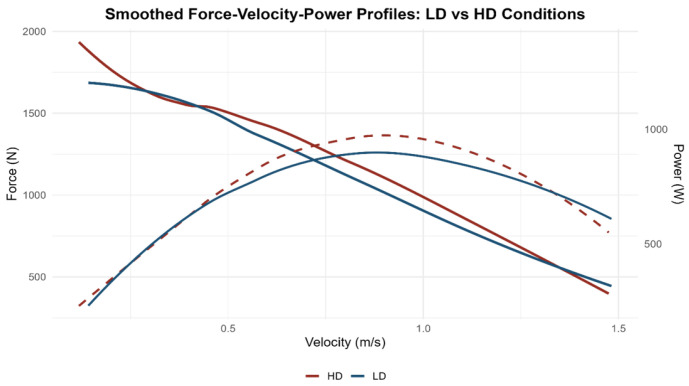
Comparison of the mean force–velocity–power (F–V–P) profiles between low-density (LD) and high-density (HD) knee sleeve conditions. Solid lines represent the smoothed force–velocity (F–v) relationships, while dashed lines depict the smoothed power–velocity (P–v) curves. Curves were obtained using LOESS smoothing (span = 0.75) to illustrate group-level trends. Force (N) is displayed on the left y-axis, power (W) on the right y-axis, and barbell velocity (m·s^−1^) on the x-axis.

The analysis of the force–velocity–power (F–V–P) profiles revealed consistent patterns across both knee sleeve conditions. The F–v curves displayed the expected inverse linear relationship between force and velocity, whereas the P–v curves showed a parabolic distribution with peak power occurring at intermediate velocities. Visual inspection indicated that the overall shapes of the curves were nearly identical between low-density (LD) and high-density (HD) sleeves, suggesting a similar neuromuscular response across conditions. Descriptive statistics for each parameter (F_0_, V_0_, Pmax, and slope) are presented in Table 2, whereas inferential statistics comparing conditions are reported in Table 3. The force–velocity–power profiles obtained under the low-density and high-density conditions are shown in Figure 1 and Figure 2, respectively. Figure 3 illustrates the mean F–V–P relationships used to compare both conditions.

A post hoc power analysis was conducted using the observed effect sizes and a paired-sample design (*n* = 15, α = 0.05). The resulting statistical power was 0.331 for F_0_, 0.277 for V_0_, 0.330 for P_max_, and 0.050 for Slope. These results indicate that the study was underpowered to detect small to moderate effects, particularly for the slope parameter, and that caution should be exercised when interpreting the nonsignificant results.

Table 2 presents the descriptive values of the variables derived from the force–velocity profile for both experimental conditions: HD and LD knee sleeves. The variables include theoretical F_0_, V_0_, P_max_, and the slope of the force–velocity relationship, which are reported using the appropriate descriptive formats according to their distributional properties (mean ± standard deviation or median [IQR]), in line with established recommendations [39,40]. In addition, 95% confidence intervals are provided for each variable.

**Table 2:** Descriptive Data of the Evaluated Conditions (LD–HD).

Descriptive Statistics by Condition				
Variable	Condition	Summary Statistic	95% CI	Reporting Format
F_0_ (N)	HD	1974.04 ± 407.30	1790.90–2157.19	Mean ± SD
V_0_ (m/s)	HD	1.96 (1.76–2.11)		Median (IQR)
P_max_ (w)	HD	873.93 (810.45–1003.32)		Median (IQR)
Slope (N × s/m)	HD	−992.08 ± 206.95	−1085.13–−899.03	Mean ± SD
F_0_ (N)	LD	1788.97 (1671.12–2023.80)		Median (IQR)
V_0_ (m/s)	LD	1.87 (1.78–1.99)		Median (IQR)
P_max_ (W)	LD	834.69 (756.19–962.95)		Median (IQR)
Slope (N × s/m)	LD	−920.05 (−1041.11–−851.57)		Median (IQR)

Abbreviations: F_0_: theoretical maximal force, V_0_: theoretical maximal velocity, P_max_: maximal power output, Slope: linear regression slope of the force–velocity profile, N: newton, W: watts, s: seconds, HD: high-density knee sleeve conditions, LD: low-density knee sleeve conditions, CI: confidence interval.

The force–velocity–power (F–V–P) profiles obtained under both knee sleeve conditions displayed the expected mechanical response pattern. The force–velocity relationship followed the characteristic inverse trend, with force progressively decreasing as movement velocity increased. In parallel, the power curve adopted a parabolic distribution, peaking at intermediate velocities and reflecting the neuromuscular capacity of the lower limbs during incremental squat actions. When comparing low- and high-density (LD and HD) conditions, the overall shape of the profiles appeared very similar, with nearly identical slopes in the force–velocity relationship. Nevertheless, a tendency towards higher absolute values of maximal force (F_0_), maximal velocity (V_0_), and maximal power (Pmax) was observed when using the higher-density sleeves. This suggests that greater material stiffness may enhance force transmission efficiency and consequently lead to a somewhat greater power output, although these differences did not reach statistical significance. Figure 1 and Figure 2 display the group mean F–V–P profiles for the LD and HD conditions, respectively, whereas Figure 3 illustrates the averaged comparison of both sleeves to highlight the minimal differences between them. Descriptive and inferential statistics for the theoretical parameters (F_0_, V_0_, Pmax, and slope) are summarised in Table 2.

**Figure 4:**
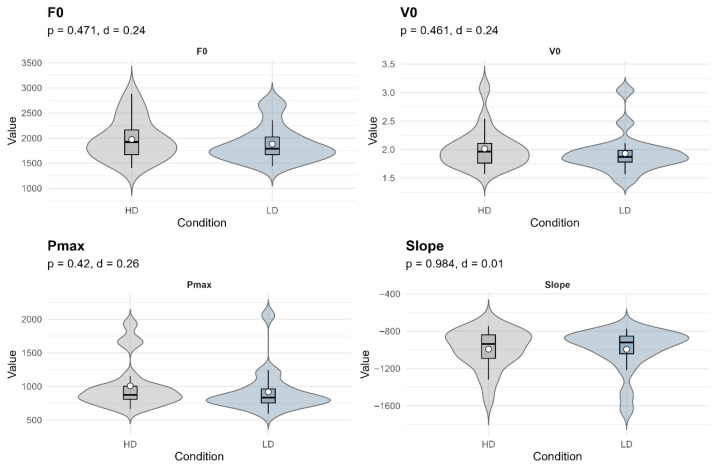
Violin plots comparing neuromuscular variables between HD and LD knee sleeve conditions. Each plot displays the distribution, median (horizontal line), interquartile range (box), and mean (white dot with standard deviation bars). The values of *p* correspond to two-tailed paired t-tests, and *d* represents Cohen’s *d* effect size. No statistically significant differences were observed in any variable. Visual dispersion and effect sizes indicate negligible to small effects across conditions.

In the comparison between conditions, significance values ranged from *p* = 0.42 to *p* = 0.98, confirming the absence of meaningful differences between HD and LD knee sleeves. Effect sizes were consistently very small across all variables: F_0_ (*d* = 0.24), V_0_ (*d* = 0.24), Pmax (*d* = 0.26), and the slope of the force–velocity relationship (*d* = 0.01). These findings indicate that, beyond the individual variability reflected in data dispersion, the use of knee sleeves with different densities did not elicit systematic changes in the neuromuscular parameters assessed. The distribution of individual responses and the absence of clear differences between conditions are illustrated in the violin plots presented in Figure 4.

Mixed-effects linear models revealed no statistically significant differences between the high-density (HD) and low-density (LD) knee sleeve conditions in any of the neuromuscular capacities evaluated.

For theoretical maximal force (F_0_), the HD condition yielded a mean of 1974 ± 407 N (95% CI: 1778 to 2170), compared to 1883 ± 359 N in the LD condition (95% CI: 1710 to 2056), with no statistically significant difference (F(1,18) = 1.30, *p* = 0.270; d = −0.37, small effect).

Theoretical maximal velocity (V_0_) was slightly higher in HD (2.01 ± 0.35 m/s; 95% CI: 1.85 to 2.18) than in LD (1.93 ± 0.35 m/s; 95% CI: 1.76 to 2.10), but this difference did not reach statistical significance (F(1,18) = 1.04, *p* = 0.321; d = −0.33, small effect).

Regarding maximal power output (Pmax), HD showed a higher mean (1010 ± 355 W; 95% CI: 839 to 1181) compared to LD (920 ± 320 W; 95% CI: 766 to 1074), without statistical significance (F(1,18) = 1.29, *p* = 0.271; d = −0.37, small effect).

Lastly, the slope of the force–velocity relationship was virtually identical across conditions: HD = −992 ± 207 N·s/m (95% CI: −1092 to −892) and LD = −994 ± 228 N·s/m (95% CI: −1103 to −884), with no statistically significant difference (F(1,18) = 0.002, *p* = 0.968; d = −0.01, trivial effect).

Although point estimates for F_0_, V_0_, and Pmax slightly favoured the HD condition, their confidence intervals crossed the null value, and the effect sizes were small or trivial, suggesting no systematic effect of sleeve type on performance.

Table 3 shows the descriptive and inferential results of the comparison of neuromuscular capacities between the high-density (HD) and low-density (LD) knee cuff conditions. Values are expressed as means ± standard deviations. The HD-LD difference reflects the absolute difference in means between conditions. The 95% confidence intervals and *p*-values correspond to linear mixed model estimates. Cohen’s d is presented as a standardised measure of effect size. No statistically significant differences were found for any variable. However, the mean differences observed in F_0_, V_0_ and Pmax slightly favoured the HD condition.

**Table 3:** Descriptive and inferential results of the comparison of neuro-muscular capacities between the high-density (HD) and low-density (LD) knee cuff conditions.

Summary of Descriptive and Inferential Results						
Variable	LD (Mean ± SD)	HD (Mean ± SD)	HD-LD Difference	95% CI	*p*-Value	Cohen’s d
F_0_ (N)	1883.3 ± 359.1	1974 ± 407.3	90.780	[−258.3, 76.73]	0.270	−0.369
V_0_ (m/s)	1.9 ± 0.3	2 ± 0.3	0.080	[−0.256, 0.089]	0.321	−0.331
P_max_ (w)	920.2 ± 320.1	1009.7 ± 354.8	89.540	[−255.18, 76.11]	0.271	−0.368
Slope (N × s/m)	−993.5 ± 228	−992.1 ± 206.9	1.440	[−76.24, 73.35]	0.968	−0.013

Abbreviations: F_0_: theoretical maximal force, V_0_: theoretical maximal velocity, P_max_: maximal power output, Slope: linear regression slope of the force–velocity profile N: newton, w: watts, s: seconds, HD: high-density knee sleeve conditions, LD: low-density knee sleeve conditions, CI: confidence interval.

From an applied perspective, the clinical significance of the observed differences was also evaluated using a minimum practical relevance threshold defined as 20% of the standard deviation of the HD condition (0.2 × SD), in accordance with Hopkins’ recommendations. In this analysis, the differences between conditions for F_0_ (90.8 N), V_0_ (0.08 m/s), and Pmax (89.5 W) exceeded their respective clinically relevant thresholds (81.5 N, 0.07 m/s, and 71.0 W, respectively), suggesting potential practical utility despite the absence of statistical significance. In contrast, the observed difference in Slope (1.4 N·s/m) was substantially below the defined threshold (41.4 N·s/m) and was therefore considered clinically trivial.

Table 4 shows how clinical significance was assessed by comparing the mean difference between conditions (HD versus LD) with a minimum significant threshold defined as 0.2 × the standard deviation of the HD condition, following Hopkins’ recommendations. Variables above this threshold were interpreted as having clinically significant differences.

Overall, no statistically significant differences were found between conditions for any of the analysed variables (*p* > 0.05), and effect sizes were small or trivial (d ≤ 0.37). Mean values of F_0_, V_0_, and Pmax were slightly higher in the high-density (HD) sleeve condition; however, their confidence intervals overlapped substantially, indicating the absence of a systematic effect of sleeve type on neuromuscular performance. These findings suggest that, while the HD sleeve may confer small practical advantages according to the 0.2 × SD criterion, such differences should be interpreted with caution given their lack of statistical significance and modest magnitude.

**Table 4:** Assessment of clinical significance by comparing the mean difference between conditions (HD versus LD) with a minimum significant threshold.

Summary of Descriptive and Inferential Results			
Variable	Difference	0.2 × SD (HD)	Interpretation
F_0_ (N)	90.78	81.46	Clinically significant
V_0_ (m/s)	0.08	0.07	Clinically significant
P_max_ (w)	89.54	70.96	Clinically significant
Slope (N × s/m)	1.44	41.39	Trivial

Abbreviations: F_0_: theoretical maximal force, V_0_: theoretical maximal velocity, P_max_: maximal power output, Slope: linear regression slope of the force–velocity profile N: newton, w: watts, s: seconds, HD: high-density knee sleeve conditions, LD: low-density knee sleeve conditions, CI: confidence interval.

In addition to the frequentist analyses, Bayesian estimation was performed to quantify the probability and magnitude of the effects between conditions. For F_0_, the Bayesian mixed model indicated a mean difference favouring the HD condition, with an 84% probability that HD > LD. However, only 48% of posterior samples exceeded the predefined threshold for clinical relevance (0.2 × SD), and approximately 50% fell within the range of trivial effects. Similarly, for Pmax, the Bayesian analysis showed an 82% probability that the HD sleeves produced higher values compared to LD, yet the probability of surpassing the minimal clinically important difference (0.2 × SD) remained below 50%, with 49% of draws classified as trivial. Together, these Bayesian results support the presence of small, directionally consistent advantages for the HD sleeves across mechanical variables, but indicate that such improvements are unlikely to be meaningfully large or practically decisive.

## 4. Discussion

The aim of this study was to determine whether the density of neoprene knee pads (HD vs. LD) has an effect on theoretical neuromuscular capacities derived from the force–velocity–power (F-V-P) profile in the motor action of the squat. Although the inferential analyses did not show statistically significant differences between the variables of maximum theoretical strength (F0), speed (V0), power output (Pmax), or the slope of the profile, there was evidence of a small favourable effect for the high-density (HD) knee pad, which exceeds the predefined thresholds of clinical relevance. From a practical point of view in performance control, this indicates that the additional compression/rigidity provided by this type of knee brace can discreetly shift the set of neuromechanical solutions towards regions of the F-V-P profile that are more favourable to sports tasks aimed at overcoming maximum and submaximal resistance (high loads and low repetitions) [41,42,43], despite having an effect size insufficient to achieve significance with the available power. In practical terms, these differences can stabilise force production in repetitions close to failure or under a large amount of accumulated fatigue, providing an operational mechanical advantage in microcycles focused on maximum force [44,45].

Despite the absence of statistically significant differences between conditions, the observed patterns can be interpreted from a biomechanical perspective. Knee sleeves of different densities may influence squat performance primarily through mechanical adaptations rather than through substantial changes in neuromuscular activation. Increased material stiffness associated with high-density sleeves may enhance joint stability, reduce soft tissue oscillation, and improve force transmission across the knee joint, thereby subtly modifying the mechanical efficiency of the movement without necessarily altering global force–velocity–power outcomes.

Within the force–velocity–power framework, such biomechanical adaptations could manifest as small shifts in theoretical parameters (e.g., F_0_, V_0_, or Pmax) without reaching statistical significance, particularly in samples with limited size. These subtle mechanical effects may become more relevant under high-load, low-velocity conditions, where external support devices can contribute to improved movement consistency and force application.

The absence of significant differences is consistent with previous scientific studies reporting heterogeneous ergogenic effects of compression and support devices in resistance training [41,46]. These devices may improve performance in maximal actions (1RM) without consistently altering the kinetics of speed or average power [10,47]. These findings suggest that the benefits of knee braces could be primarily mechanical: greater joint stability, greater proprioception, and less segmental oscillation [20,48,49]. These benefits may not necessarily be due to global changes in neuromuscular activation [50]. In the F-V-P profile, the slight increases provided by HD in F0, V0, and Pmax are consistent with greater effective stiffness of the system (material and soft tissue), which would improve force production and transmission, reducing losses due to deformation [20,48]. An accumulation and release of elastic energy during the transition at the sarcomere level in the eccentric and concentric contractile phases, facilitating concentric power [51,52]. And better postural control, which according to various authors would reduce micro-misalignments and coordination costs [53]. However, these effects could be attributed to expectation or perceived safety (placebo) [54], Therefore, future research should blind expectations and standardise the adjustment to dissociate these material effects from perceptual ones.

Although statistical analyses did not show significant differences (*p* > 0.005), improvements in F_0_ (+90.8 N), V_0_ (+0.08 m·s^−1^) and Pmax (+89.5 W) exceeded the 0.2 × SD threshold for clinical relevance, suggesting small but useful effects [55,56,57]. Complementing these observations, the Bayesian analyses provided additional insight into the magnitude and practical meaning of these effects. For both F0 and Pmax, the posterior distributions indicated moderate probabilities (≈82–84%) that the HD condition produced higher values than LD, supporting the directional consistency of the effects. However, the probability that these improvements surpassed the minimal clinically important difference (0.2 × SD) remained below 50%, and approximately half of the posterior draws fell within the region of trivial effects. This Bayesian evidence reinforces the interpretation that the advantages of HD sleeves, although mechanically plausible and directionally stable, are small in magnitude and unlikely to reflect a robust or systematic improvement across athletes. Rather than contradicting the frequentist findings, the Bayesian results add nuance by quantifying the uncertainty and highlighting that the observed improvements, while real in direction, remain modest and context dependent.

In disciplines (powerlifting, weightlifting or strength mesocycles within team sports) where these minimal margins determine the outcome, even a slight gain in mechanical efficiency can shift the F-V-P profile towards regions of greater force generation at low speeds, as well as improving repeatability between sets (lower intra-subject variability) and sustain power in blocks with high neuromuscular stress [41,42]. Considering that neoprene knee pads report modest benefits that depend on the sporting context, selective use of the HD option seems to be optimal for high-load, low-repetition sessions or on key days (testing, peak preparation) [58,59]. These recommendations are consistent with previous studies reporting improvements in 1RM in squats without clear changes in speed or power when using neoprene knee pads, a pattern consistent with primarily mechanical rather than neuromuscular mechanisms [9,24]. Studies analysing other devices such as belts and straps reported kinematic improvements in execution time and lower RPE during maximum effort, reinforcing the situational usefulness of external supports in sports tasks with high energy and mechanical demands [10]. In parallel, a meta-analysis on compression garments reports a small and heterogeneous effect depending on the task and pressure dosage, which highlights the need to generate individual prescriptions for each athlete [22]. To interpret these small but potentially useful changes, it is suggested to report confidence intervals (CI), effect sizes (Hedges) and, when necessary, a test of equivalence (TOST) to distinguish whether ‘non-significance’ would imply practical equivalence or insufficient power in small samples.

This research contributes to the understanding of strength-oriented athletic performance by shifting the assessment from discrete metrics (1RM and jump height) to a continuous F-V-P profile framework, which integrates information on strength, speed, and power across the entire load spectrum [60,61]. The combination of linear adjustments with LOESS smoothing allows us to finely read the neuromuscular organisation and efficiency in force transmission and generation, demonstrating that the compressive effects generated by knee pads of different densities can manifest as subtle shifts in the complete profile (F-V-P), despite not presenting significant differences [60,62]. When interpreting these data, the pattern observed and described (small improvements with HD that exceed criteria of clinical relevance) is consistent with mainly mechanical mechanisms [20,48] and with an optimisation of eccentric-concentric exchange, which favours force production at low speeds [63,64]. Finally, the F-V-P profile used in this study adds resolution to distinguish potentially useful changes in contexts where high mechanical demand is required [60,65,66].

Building upon these insights, it is essential to acknowledge certain methodological and practical considerations that may guide future applications and research.

### 4.1. Limitations

Due to the nature of the study, several methodological limitations should be acknowledged. The sample was small and homogeneous, comprising only young recreationally trained men, which limits the generalizability of the findings to other populations such as elite athletes or female participants. Moreover, the density of the sleeves was classified as high versus low without direct measurement of compression pressure (mmHg) or material stiffness, precluding a dose–response analysis of mechanical effects. Blinding of participants was not possible due to the visible differences between sleeves, which could have influenced performance expectations. Finally, the use of group-level linear and LOESS analyses provides a robust overview but does not capture potential individual responses, which may vary meaningfully in applied contexts. Future research should therefore include larger and more diverse samples, quantify compression parameters, and integrate electromyographic and 3D kinematic measures to better elucidate the underlying neuromechanical mechanisms.

A limitation of the present study is the absence of direct measurements of muscle activity. The inclusion of electromyographic (EMG) parameters in future research could help clarify whether knee sleeve density influences muscle activation patterns, intermuscular coordination, or co-contraction strategies during the squat. Such measures would allow differentiation between purely mechanical effects of sleeve stiffness and potential neuromuscular adaptations associated with perceived stability or proprioceptive feedback.

### 4.2. Applicability and Future Research

These results do not justify changing future training prescriptions solely based on the density of the knee pads; however, the use of high-density knee pads is recommended for selective occasions, such as high-load, low-repetition sessions, provided that they are individually adjusted and do not compromise movement technique. In addition, it is recommended to interpret performance changes using confidence intervals and effect sizes, and to monitor athletes’ progress through force–velocity–power (F–V–P) profiling. Overall, high-density sleeves may offer small but potentially meaningful advantages in contexts where high mechanical demand or maximal strength expression is required, although these effects should be considered complementary rather than decisive in training decision-making.

## 5. Conclusions

In inferential terms, the density of the knee pads (HD and LD) did not provide a statistically significant improvement in the theoretical neuromuscular capacities derived from the F–V–P profile in the squat. However, consistent but small differences were observed in favour of the HD knee pad, which exceeded the predefined thresholds of clinical relevance for F_0_, V_0_ and Pmax, suggesting possible practical implications in strength-oriented contexts, especially when the mechanical task approaches maximum demands (high loads and low speeds). Bayesian analyses further supported this interpretation, showing moderate probabilities that the HD condition produced higher values than LD across key mechanical variables (≈82–84%), but with less than a 50% probability that these increases surpassed the minimum clinically important difference and a substantial portion of the posterior distributions falling within the range of trivial effects. Taken together, these findings reinforce the value of the F–V–P profile as an analytical framework capable of detecting subtle shifts in athlete performance that might go unnoticed when traditional metrics are used, while indicating that the advantages provided by HD knee pads, although directionally consistent, are likely small and context dependent.

## Data Availability

The original contributions presented in this study are included in the article. Further inquiries can be directed to the corresponding author.
